# Successful diaphragm repair following radiofrequency ablation for renal cell carcinoma: A case report

**DOI:** 10.1016/j.ijscr.2024.110371

**Published:** 2024-09-30

**Authors:** Wenxue Piao, Sung Joon Han

**Affiliations:** Department of Thoracic and Cardiovascular Surgery, Chungnam National University Hospital, Republic of Korea

**Keywords:** Traumatic diaphragmatic rupture, Radiofrequency ablation, Renal cell carcinoma

## Abstract

**Introduction and importance:**

This study aimed to assess the effectiveness of surgical intervention in treating traumatic diaphragmatic rupture accompanied by pleural empyema resulting from radiofrequency ablation for renal cell carcinoma.

**Case presentation:**

A 72-year-old female patient underwent radiofrequency ablation at our institution's urology department to address a 4-cm tumor in the left upper kidney detected during routine health screening. Subsequently, the patient experienced persistent fever from the 5th day post- procedure. Chest radiography revealed increased opacity in the left lower lung, prompting further evaluation with contrast-enhanced chest computed tomography. Examination revealed multiple loculated effusions and discernible diaphragmatic defects. Consequently, the patient was referred to the department of thoracic surgery, where an emergency surgery was performed. The surgical procedure was performed under general anesthesia the following day, revealing a 4-centimeter defect in the diaphragm along with damaged surrounding tissue and multiple loculated empyema sacs within the thoracic cavity. The intervention included excision of the empyema sacs, extensive irrigation, and reconstruction of the diaphragm using a 2-mm Gore- Tex membrane. One week postoperatively, the patient was discharged without any complications related to the procedure.

**Clinical discussion:**

Although radiofrequency ablation is considered a relatively safe procedure with low complication rates, vigilant post-procedural monitoring is essential for detecting potentially serious complications.

**Conclusion:**

Surgical intervention remains the preferred approach for the repair of traumatic diaphragmatic ruptures and is typically performed via thoracotomy.

## Highlights

• We treated traumatic diaphragmatic rupture+pleural empyema due to RFA for RCC.
• This case serves as a reminder of the potential adverse events of RFA for RCC.
• Effective postprocedural monitoring and prompt surgical intervention are essential.

## 1 Introduction and importance

Renal cell carcinoma (RCC) is a common type of kidney cancer in adults. The incidence of RCC varies geographically and is increasing worldwide. In the United States, RCC accounts for approximately 3–4 % of all adult malignancies. The American Cancer Society estimates that there will be approximately 76,080 new cases of kidney cancer, including renal pelvis and ureter cancers, in the United States in 2022, with approximately 62,710 of these cases being RCC specifically. Globally, the incidence of RCC varies by region, with higher rates reported in developed countries than in developing countries. In Europe, RCC is estimated to account for approximately 2–3 % of all adult cancers. The incidence rates of RCC have been rising in many countries, partly due to improvements in diagnostic techniques leading to increased detection of small renal tumours, as well as changes in lifestyle factors, including smoking, obesity, and hypertension [1].

RCC presents a complex clinical challenge that necessitates continuous advancements in diagnostic methodologies and therapeutic approaches. Treatment options for RCC vary depending on factors such as cancer stage, tumor size and location, patient health, and preferences [2]. Surgical intervention, including radical or partial nephrectomy, remains the cornerstone of localized RCC [3]. Alternatively, for advanced or metastatic diseases, targeted therapy utilizing drugs such as tyrosine kinase inhibitors and immunotherapy, including checkpoint inhibitors such as pembrolizumab and nivolumab, are commonly employed. Radiation therapy, chemotherapy, and minimally invasive techniques, such as radiofrequency ablation (RFA) or cryoablation, may also be utilized in certain cases.

Moreover, participation in clinical trials investigating novel treatment approaches offers potential avenues for patients with RCC. Treatment decisions are typically collaborative and involve a multidisciplinary team of specialists who tailor personalized treatment plans to each patient's unique circumstances [[4], [5], [6]].

RFA is a minimally invasive procedure that uses the heat generated by radiofrequency energy to destroy cancer cells. It is typically considered a treatment option for RCC in cases where the tumor is small (usually less than 3–4 cm in diameter) and localized to the kidney.

Diaphragmatic injury resulting from RFA is an infrequent yet possible complication of this procedure [7]. Meiqi Zhou et al. reported that thoracic irrigation combined with simple suturing of the diaphragmatic defect is an effective method for managing RFA-induced diaphragmatic injury. This report emphasizes the need for early detection and timely surgical intervention, as in our case, to ensure favorable patient outcomes. RFA entails the application of heat generated by radiofrequency energy to eradicate targeted tissues, usually tumours. Although generally considered a safe and efficient minimally invasive intervention, there is a risk of inadvertent harm to neighbouring structures, such as the diaphragm, particularly if the tumor is closely situated or in direct contact with it. Various mechanisms can lead to diaphragmatic injury during RFA, including heat transfer, direct trauma from instruments, energy dispersion, and visualization challenges [8]. To mitigate this risk, meticulous preprocedural planning and imaging assessments are crucial to evaluate the proximity of tumours to critical structures. Real-time monitoring and precise electrode placement during the procedure, aided by imaging modalities such as ultrasonography or CT, are essential to minimize the likelihood of complications [9]. Despite these precautions, diaphragmatic injuries remain a potential hazard, underscoring the need for vigilant monitoring and expertise in the management of such occurrences.

Herein, we present a case report detailing the successful repair of the diaphragm following RFA of RCC.

## 2 Case presentation

**Fig. 1:**
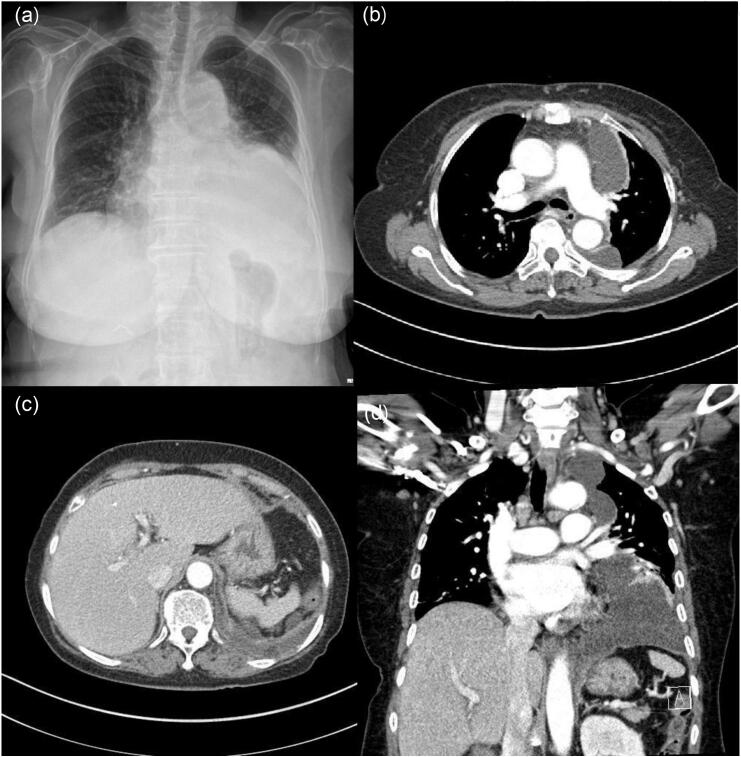
(a) Chest radiograph shows increase in amount of left pleural effusion with consolidation and atelectasis in left lower zone. (b–d) show multilobulated pleural effusion in left hemithorax and left diaphragmatic defect.

A 72-year-old female patient underwent RFA at our institution's urology department to address a 4-cm tumor in the left upper kidney detected during routine health screening. Although partial nephrectomy is a common treatment for renal cell carcinoma of this size (4 cm), in this case, RFA was chosen due to the patient's age and underlying comorbidities. These factors made a less invasive approach more suitable to minimize surgical risks. Subsequently, the patient experienced persistent fever from the 5th day post-procedure. Chest radiography revealed increased opacity in the left lower lung, prompting further evaluation with contrast-enhanced chest Computed Tomography (Fig. 1). Examination revealed multiple loculated effusions and discernible diaphragmatic defects. Consequently, the patient was referred to the department of thoracic surgery, where an emergency surgery was performed.

**Fig. 2:**
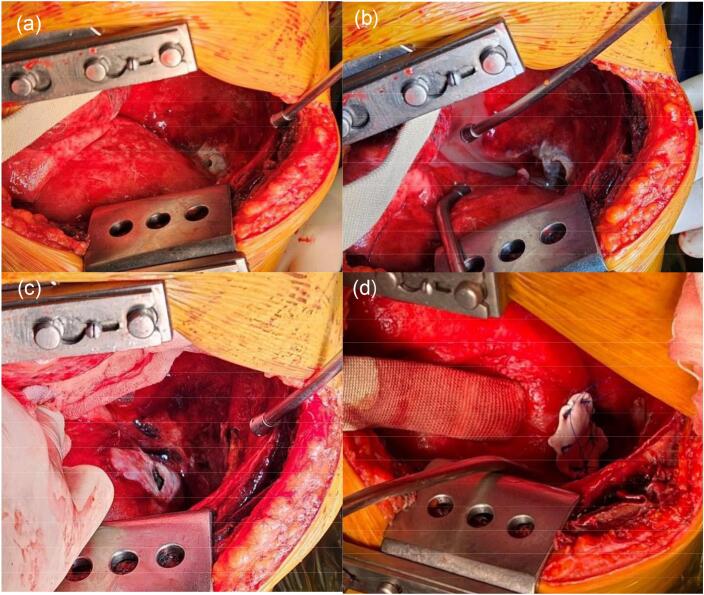
(a–c) Intraoperative images of a detected hole with fluid from the abdomen after retraction of left lung. (d) shows successful diaphragm reconstruction with Gore-Tex membrane.

The surgical procedure, conducted under general anesthesia the following day, revealed a visible 4-cm defect in the diaphragm along with damaged surrounding tissue. Additionally, multiple loculated empyema sacs were identified in the thoracic cavity. Intervention included excision of the empyema sacs, extensive irrigation, and reconstruction of the diaphragm using a 2-mm Gore-Tex membrane (Fig. 2). One week postoperatively, the patient was discharged without any complications related to the procedure.

**Fig. 3:**
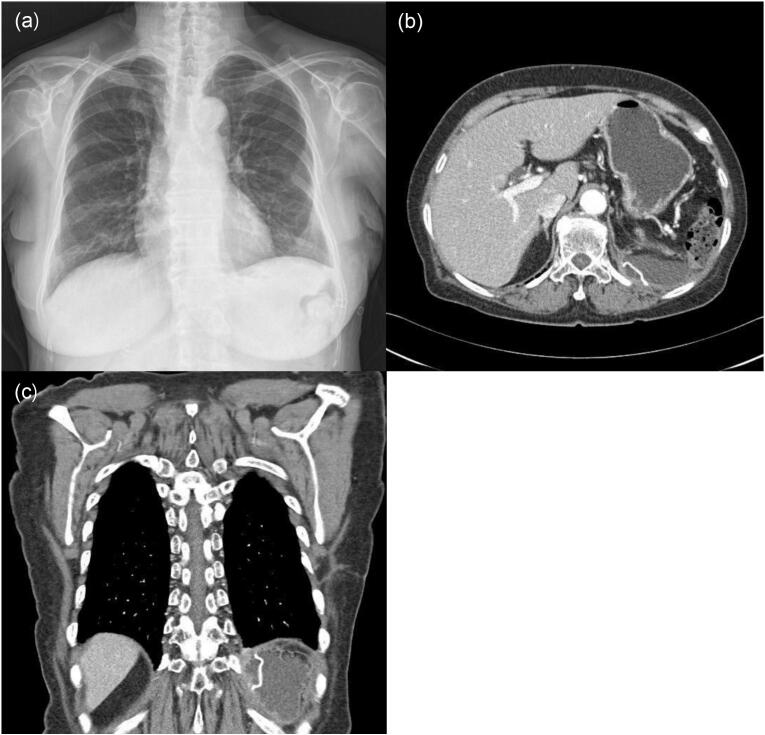
(a) Disappearance of the previous pleural effusion with consolidation in the lower left zone. (b) and (c) show the preserved operation site of the empyemectomy and repair of the diaphragmatic defect with the Gore-Tex membrane.

Two months postoperatively, a follow-up chest CT scan revealed a clear operation site for empyemectomy and repair of the diaphragmatic defect with no definite related complications (Fig. 3).

The work has been reported in line with the SCARE criteria [10].

## 3 Clinical discussion

This case highlights the importance of postprocedural vigilance in patients undergoing RFA for RCC. Despite the established safety profile of RFA, our findings underscore the potential for severe complications such as traumatic diaphragmatic rupture accompanied by pleural empyema, necessitating prompt and effective surgical intervention. During RFA, complications such as diaphragmatic injury can occur due to mechanisms like heat transmission to adjacent tissues, direct trauma from instruments, or improper electrode placement, particularly when the tumor is located near the diaphragm. These risks underscore the need for meticulous preprocedural imaging and intraoperative monitoring to minimize the likelihood of such adverse events.

Our case demonstrates that even minimally invasive procedures such as RFA can lead to significant post-procedural complications. The identification of a 4-cm diaphragmatic defect and multiple loculated empyema sacs emphasized the potential severity of such complications. The immediate surgical response, involving repair of the diaphragm with a Gore-Tex membrane and removal of the empyema sacs, was crucial in ensuring positive outcomes for the patient. This suggests that timely surgical intervention is vital for the effective management of such adverse events.

The success of surgical intervention in this case supports the efficacy of thoracotomy in addressing traumatic diaphragmatic ruptures. The use of a Gore-Tex membrane for diaphragmatic reconstruction provides durable repair, which is essential, given the extent of tissue damage [11]. The patient's recovery, characterized by discharge without procedural complications one week postoperatively, further underscores the benefits of a well- coordinated surgical approach. This case also illustrates the necessity for rigorous post- procedural monitoring. The patient's persistent fever from the fifth day post-RFA was a critical indicator of underlying complications, prompting further diagnostic imaging. The roles of chest radiography and contrast-enhanced CT in diagnosing diaphragmatic rupture and pleural empyema are pivotal, demonstrating the value of these imaging modalities in the early detection of serious post-RFA complications [12,13].

Previous literature on complications of radiofrequency ablation has focused on common problems such as thermal injury to adjacent organs and post-ablation syndrome (which usually includes fever and malaise). Wah et al. conducted a prospective investigation of post-radiofrequency ablation syndrome, in which patients presented with low-grade fever and flu-like symptoms that resolved spontaneously within 10 days of the procedure [14]. However, cases of diaphragmatic rupture are rare and highlight potentially life-threatening complications requiring immediate surgical management [15]. Our findings are consistent with those of other reports emphasizing the need for heightened awareness and prompt surgical referral when severe complications arise.

In addition to the surgical aspects, this case highlights the broader implications of RFA in clinical practice for the management of RCC, demonstrating the need for multidisciplinary teams involving interventional radiologists, thoracic surgeons, and intensive care specialists to collaborate closely and ensure comprehensive care from the initial procedure through to postoperative recovery [16]. The integration of such teams can facilitate the early recognition of complications and expedite necessary interventions.

This case study suggests potential avenues for future research in this area. Investigating the incidence of severe complications in larger cohorts could provide valuable data to refine patient selection criteria and procedural protocols. Identifying predisposing factors for diaphragmatic rupture and pleural empyema could help stratify patients according to risk and tailor follow-up protocols. Such research could also explore the optimization of post-RFA imaging schedules to ensure early detection and intervention of complications, potentially improving patient outcomes.

Furthermore, patient education plays a critical role in post-procedural care. Ensuring that patients are well informed about the signs and symptoms of potential complications can lead to earlier self-reporting and medical consultation, facilitating timely intervention [17]. This case highlights the importance of detailed discharge instructions and the need for robust communication channels between patients and healthcare providers.

In conclusion, although RFA remains a valuable and minimally invasive option for the treatment of RCC, this case underscores the necessity for vigilant post-procedural monitoring and readiness to address severe complications surgically. Successful management of traumatic diaphragmatic rupture with pleural empyema through timely surgical intervention highlights the importance of a multidisciplinary approach and offers insights for improving clinical practice and patient outcomes. Further research and patient education initiatives are essential to enhance the safety and effectiveness of RFA for the treatment of RCC.

## 4 Conclusion

Although RFA remains a valuable treatment option for RCC owing to its minimally invasive nature and low complication rates, this case serves as a reminder of its potential for serious adverse events. Effective postprocedural monitoring and prompt surgical intervention are essential to manage complications such as diaphragmatic rupture and pleural empyema, ensuring favorable patient outcomes. Future research should continue to explore strategies for early detection and management of RFA complications to further enhance patient safety and care quality.

## Ethical approval

This study is exempt from ethical approval as it is conducted under the condition of patient's consent.

## Declaration of Generative AI and AI-assisted technologies in the writing process

In preparing this manuscript, the authors utilized OpenAI's ChatGPT to assist with drafting and refining certain sections of the text. The final content has been reviewed and approved by all authors to ensure accuracy and alignment with the study's objectives.

## Funding

No funding.

## Author contribution

Wenxue Piao: Conceptualization, Methodology, Writing - Original Draft

Sung Joon Han: Investigation, Visualization, Project Administration, Supervision

## Guarantor

Sung Joon Han

## Research registration number

N/A

## Ethics statement

This study was conducted with informed consent obtained from individual participants included in the study.

## Consent

Written informed consent was obtained from the patient for publication and any accompanying images. A copy of the written consent is available for review by the Editor-in-Chief of this journal on request (Chungnam National University Hospital, Republic of Korea).

## Conflict of interest statement

No conflicts of interest related to this work.

## References

[1] Znaor A., Lortet-Tieulent J., Laversanne M., Jemal A., Bray F. (2015). International variations and trends in renal cell carcinoma incidence and mortality. Eur. Urol..

[2] Barata P.C., Rini B.I. (2017). Treatment of renal cell carcinoma: current status and future directions. CA Cancer J. Clin..

[3] Ljungberg B., Bensalah K., Canfield S., Dabestani S., Hofmann F., Hora M. (2015). EAU guidelines on renal cell carcinoma: 2014 update. Eur. Urol..

[4] Gilligan T., Lin D.W., Aggarwal R., Chism D., Cost N., Derweesh I.H. (2019). Testicular cancer, version 2.2020, NCCN clinical practice guidelines in oncology. J. Natl. Compr. Canc. Netw..

[5] Rini B.I., Plimack E.R., Stus V., Gafanov R., Hawkins R., Nosov D. (2019). Pembrolizumab plus axitinib versus sunitinib for advanced renal-cell carcinoma. New Engl J Med.

[6] Choueiri T.K., Motzer R.J. (2017). Systemic therapy for metastatic renal-cell carcinoma. New Engl J Med.

[7] Park B.K., Kim C.K. (2009). Complications of image-guided radiofrequency ablation of renal cell carcinoma: causes, imaging features and prevention methods. Eur. Radiol..

[8] Schullian P., Putzer D., Laimer G., Levy E., Bale R. (2020). Feasibility, safety, and long-term efficacy of stereotactic radiofrequency ablation for tumours adjacent to the diaphragm in the hepatic dome: a case-control study. Eur. Radiol..

[9] Lee Y., Yoon J.H., Han S., Joo I., Lee J.M. (2024). Contrast-enhanced ultrasonography–CT/MRI fusion guidance for percutaneous ablation of inconspicuous, small liver tumours: improving feasibility and therapeutic outcome. Cancer Imaging.

[10] Sohrabi C., Mathew G., Maria N., Kerwan A., Franchi T., Agha R.A. (2023). The SCARE 2023 guideline: updating consensus surgical CAse REport (SCARE) guidelines. Int J Surg Lond Engl..

[11] Kim H.K., Kim W.H., Kim S.C., Lim C., Lee C.H., Kim S.J. (2006). Surgical strategy for pulmonary coarctation in the univentricular heart. Eur. J. Cardiothorac. Surg..

[12] Hammer M.M., Raptis D.A., Mellnick V.M., Bhalla S., Raptis C.A. (2017). Traumatic injuries of the diaphragm: overview of imaging findings and diagnosis. Abdom Radiol (NY).

[13] Thiam O., Konate I., Gueye M.L., Toure A.O., Seck M., Cisse M. (2016). Traumatic diaphragmatic injuries: epidemiological, diagnostic and therapeutic aspects. Springerplus.

[14] Wah T.M., Arellano R.S., Gervais D.A., Saltalamacchia C.A., Martino J., Halpern E.F. (2005). Image-guided percutaneous radiofrequency ablation and incidence of post–radiofrequency ablation syndrome: prospective survey. Radiology.

[15] Kim J.S., Kim H.S., Myung D.S., Lee G.H., Park K.J., Cho S.B. (2013). A case of diaphragmatic hernia induced by radiofrequency ablation for hepatocellular carcinoma. Korean J. Gastroenterol..

[16] European Society of Radiology (ESR) (2023). “Role of radiology in a multidisciplinary approach to patient care”: summary of the ESR international forum 2022. *Insights*. Imaging.

[17] Gillespie B.M., Thalib L., Harbeck E., Tobiano G., Kang E., Tobiano S. (2023). Effectiveness of discharge education for patients undergoing general surgery: a systematic review and meta-analysis. Int. J. Nurs. Stud..

